# Supplementary material for: Vapor detection and discrimination with a panel of odorant receptors
Source: Nat Commun. 2018 Nov 1;9:4556. doi: 10.1038/s41467-018-06806-w (PMC6212438; doi:10.1038/s41467-018-06806-w)
Supplement: Supplementary file 1 — Supplementary Information [file 41467_2018_6806_MOESM1_ESM.pdf]

Supplementary information

Vapor detection and discrimination with a panel of odorant receptors

Kida et. al.

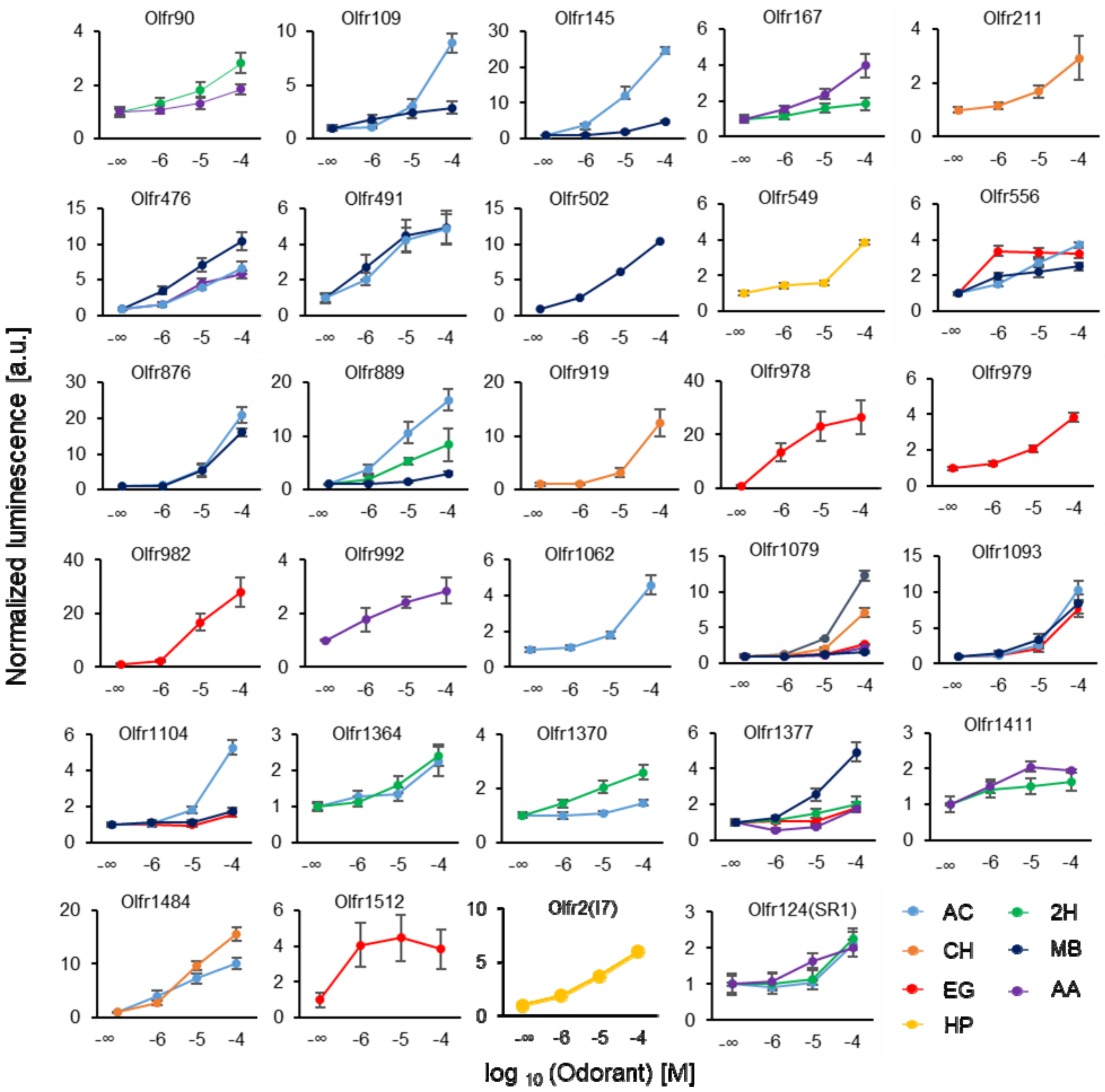

Supplementary Fig. 1
Results of secondary screening of 29 ORs used in this research. Responses were normalized such that response of each receptor at no odor condition was defined 1. Error bars indicate s.e.m. (n=3).

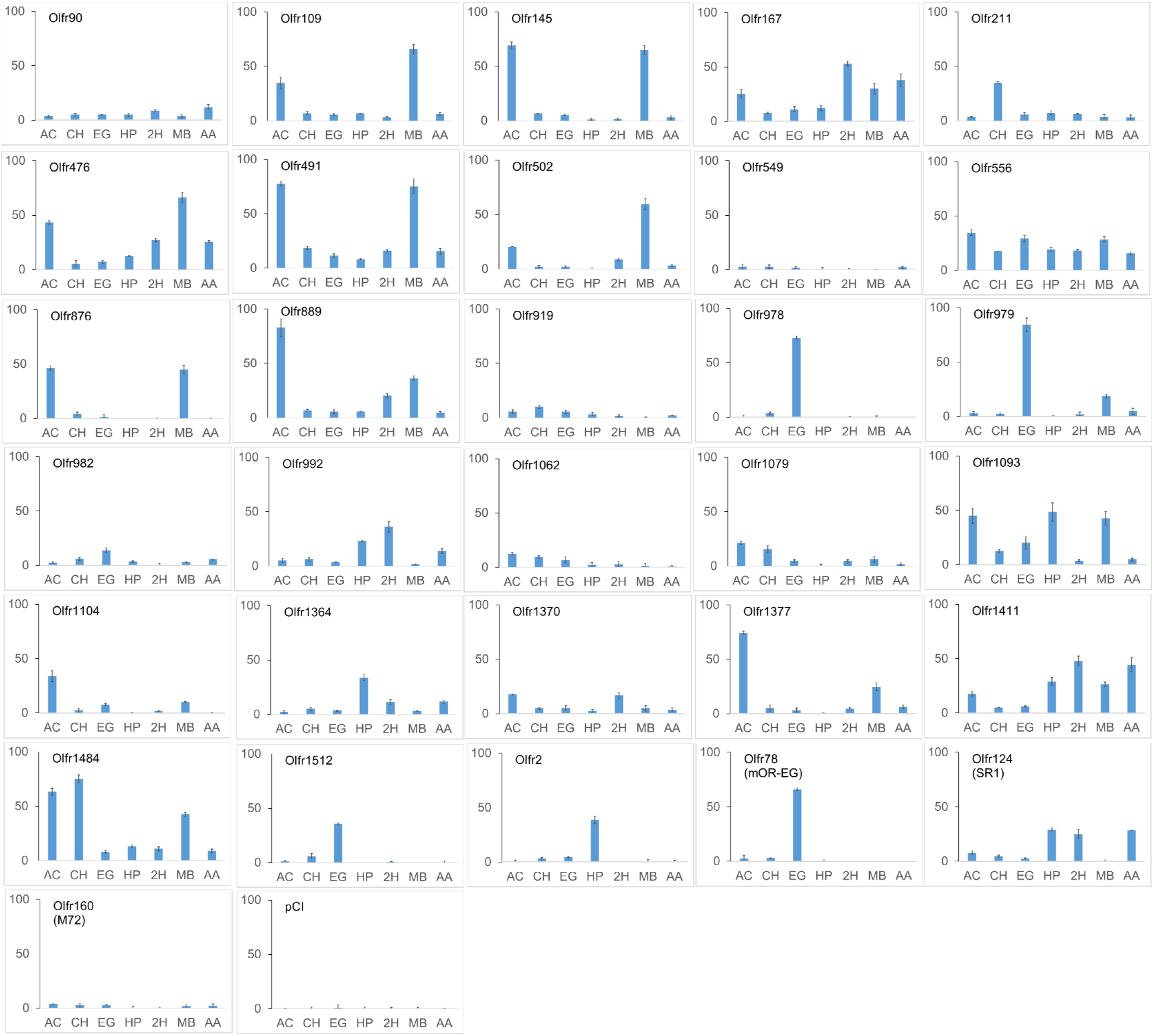

Supplementary Fig. 2
AUC of each OR with 50 uM of 7 odorants by liquid stimulation.
Error bars indicate s.e.m. (n=3).

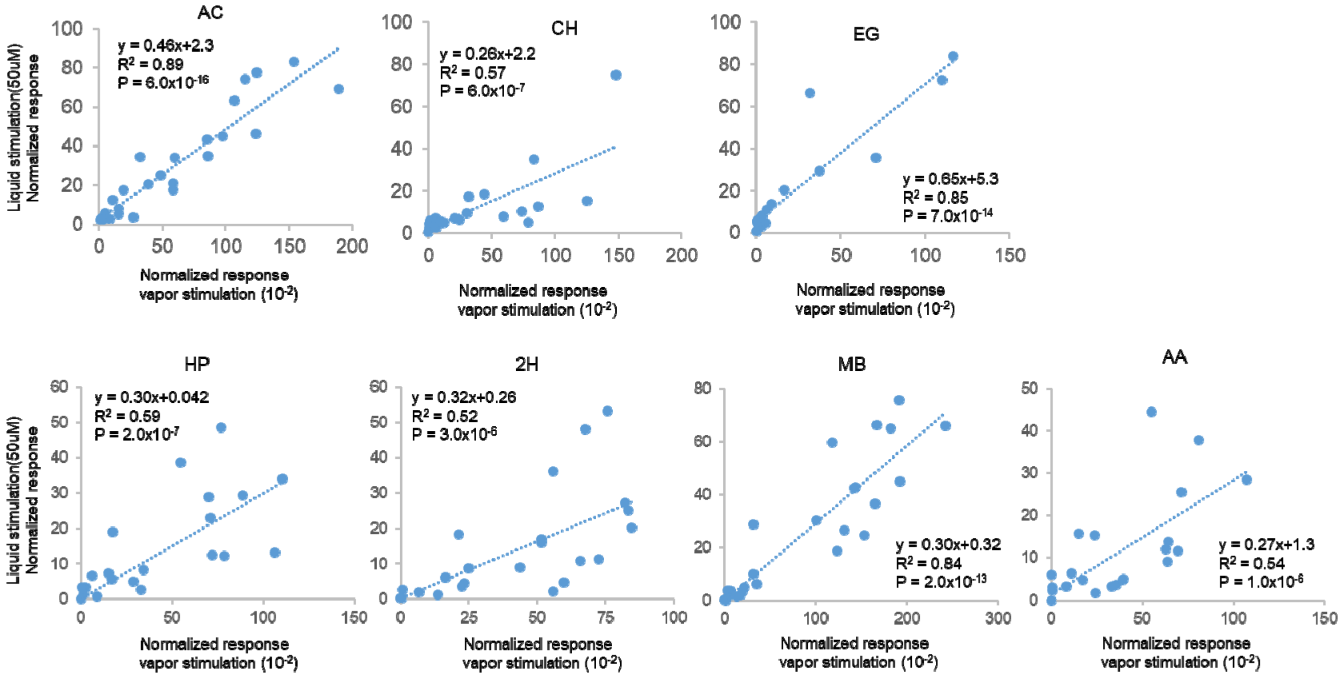

Supplementary Fig. 3

Comparison of vapor and liquid stimulation in 7 odorants. The AUC with vapor was stimulated with 10^{-2}. The AUC value with liquid was directly stimulated with 50uM dilution. The straight line is the regression line and R^2 indicates Pearson's correlation coefficient. The p-value was calculated by a t-test.

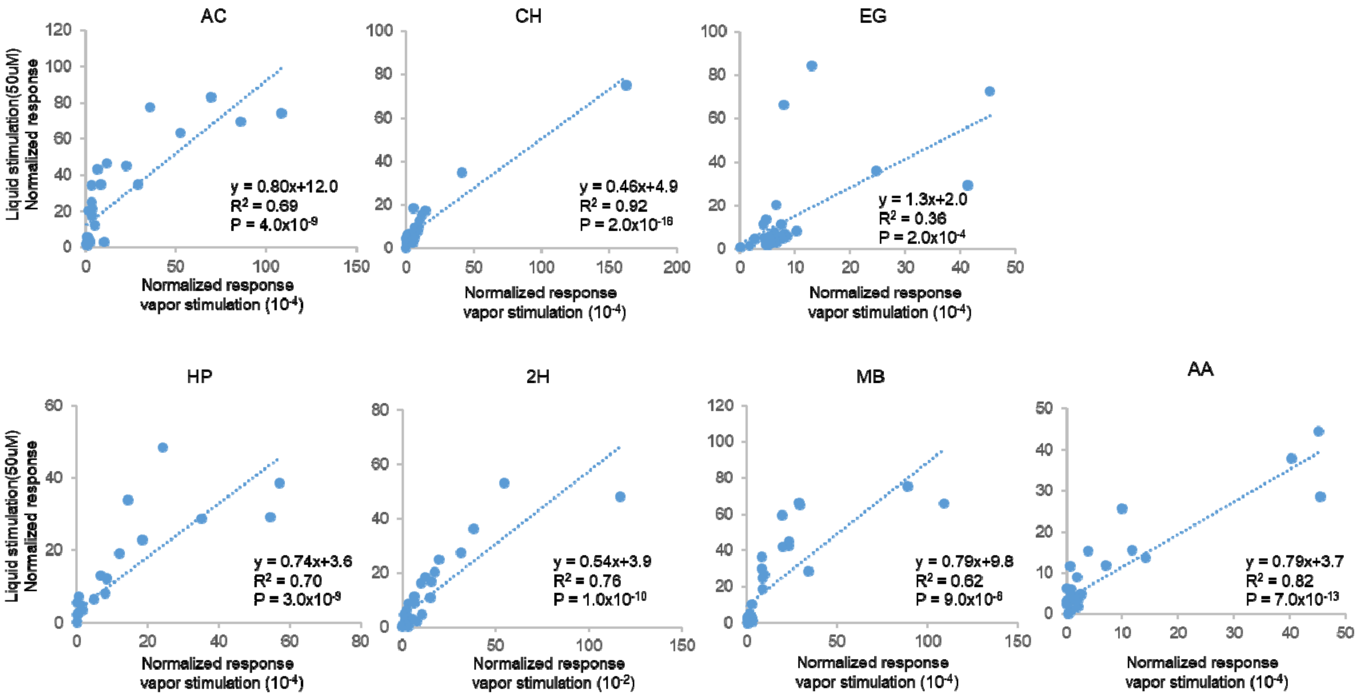

Supplementary Fig. 4

Comparison of vapor and liquid stimulation in 7 odorants. The AUC with vapor was stimulated with 10^{-4}. The AUC value with liquid was directly stimulated with 50uM dilution. The straight line is regression line and R^2 indicates Pearson's correlation coefficient. The p-value was calculated by t-test.

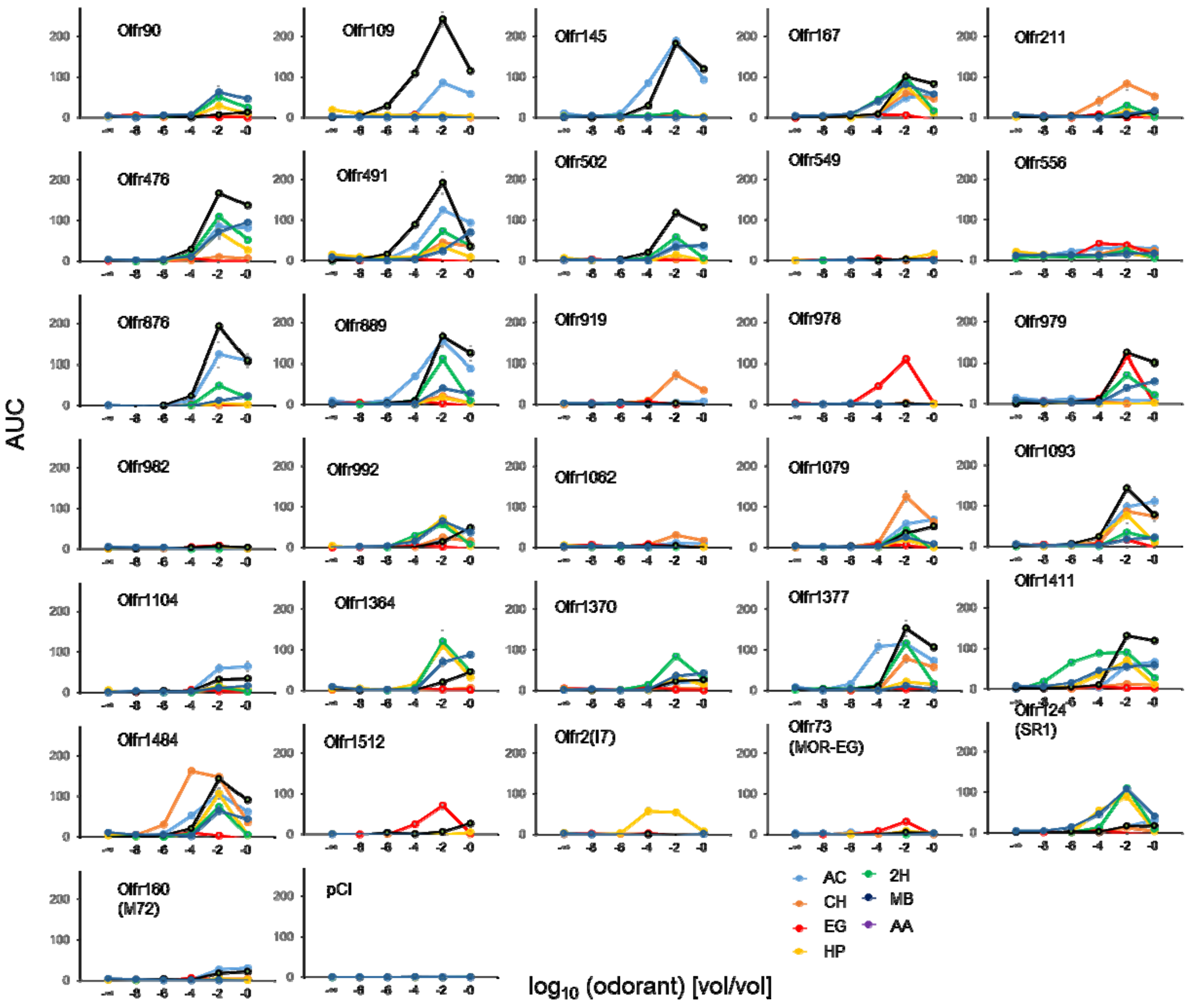

Supplementary Fig. 5

Dynamic range of ORs with each odorant by vapor stimulation.

Error bars indicate s.e.m. (n=3).

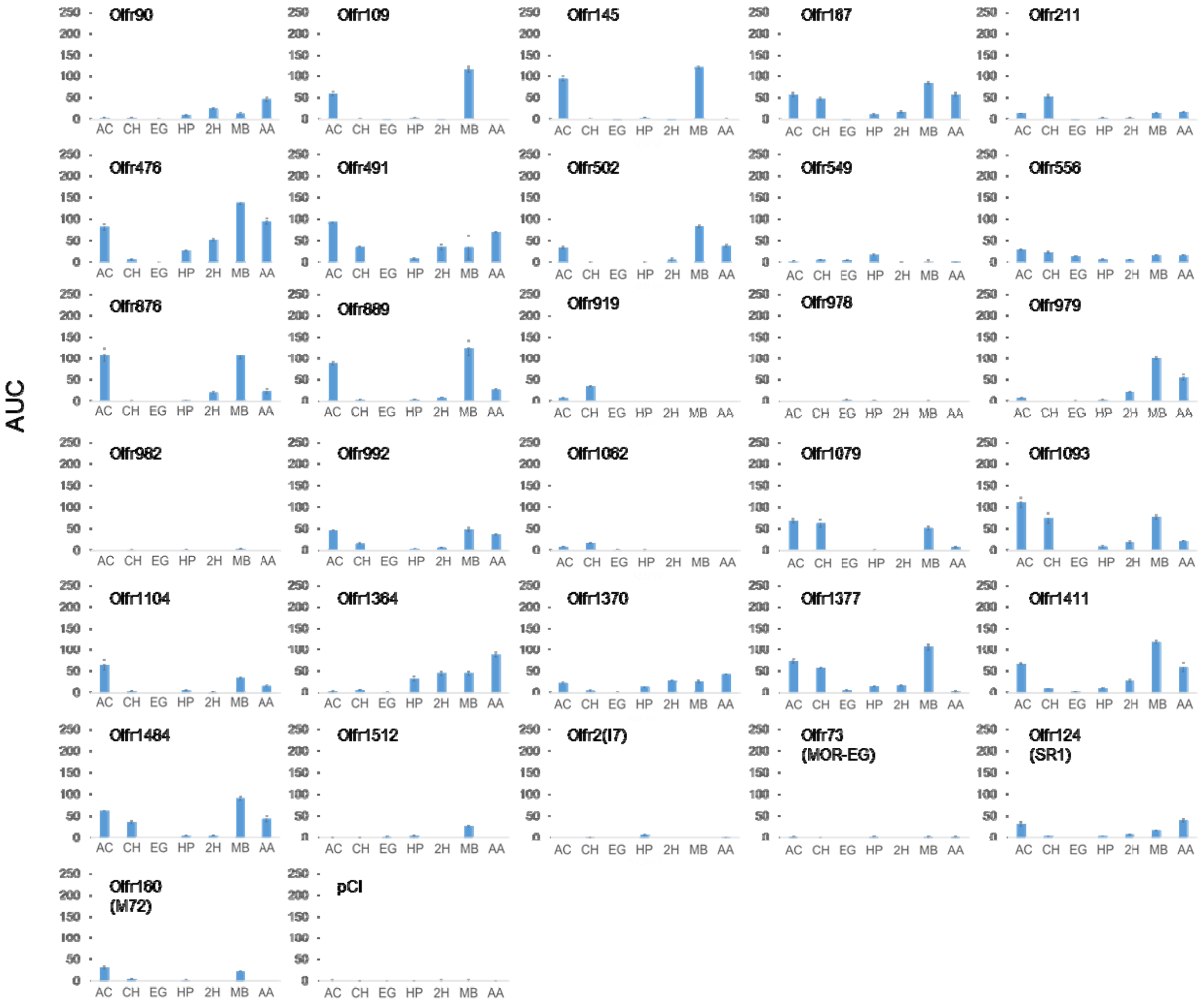

Supplementary Fig. 6
AUC of each OR with stimulated with an undiluted odorant. Error bars show s.e.m.(n=3).

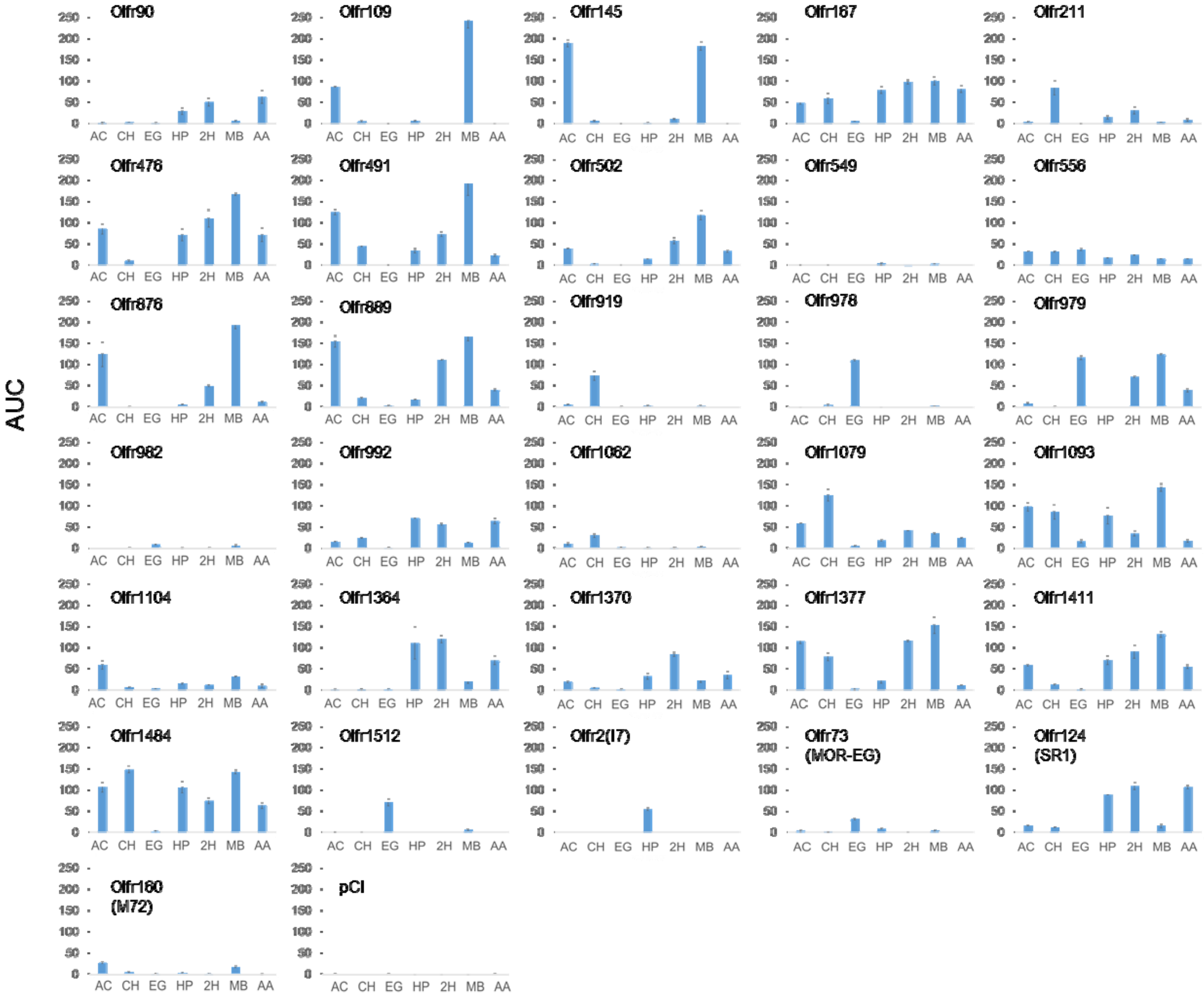

Supplementary Fig. 7
AUC of each OR with stimulated with an odorant at 10^{-2} dilution.
Error bars show s.e.m.(n=3).

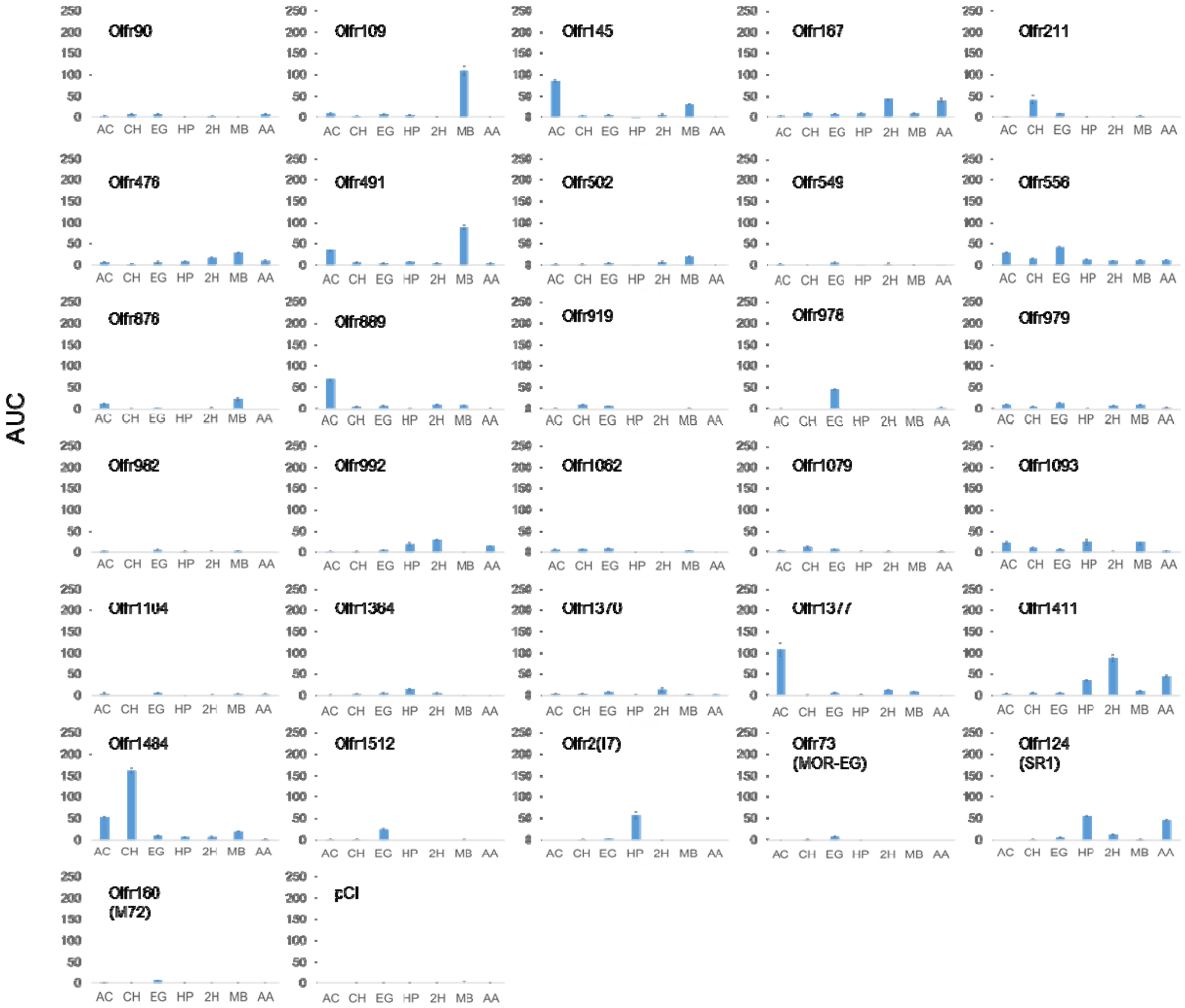

Supplementary Fig. 8

AUC of each OR with stimulated with an odorant at 10^{-4} dilution.

Error bars show s.e.m.(n=3).

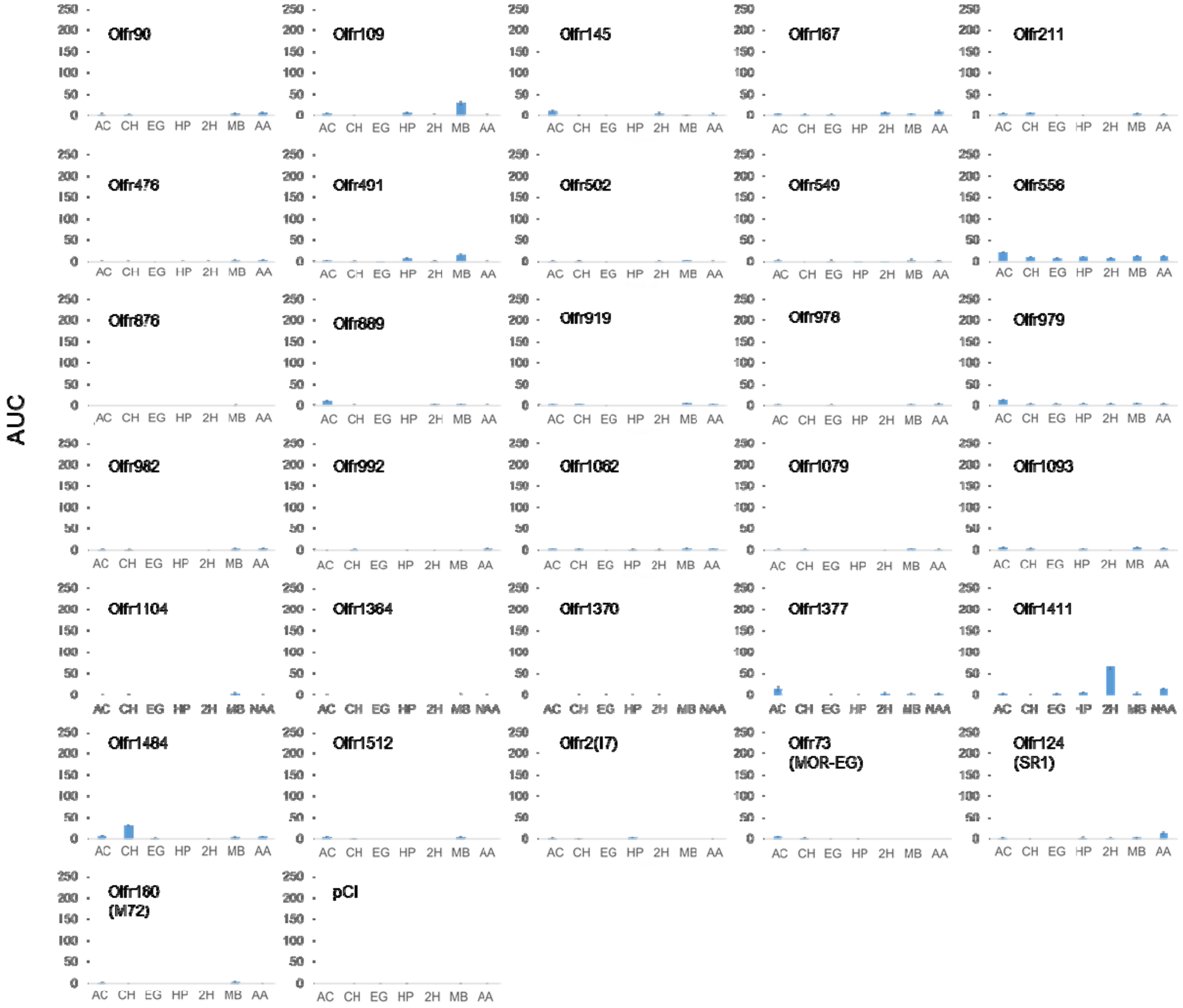

Supplementary Fig. 9
AUC of each OR with stimulated with an odorant at 10^{-6} dilution.
Error bars show s.e.m.(n=3).

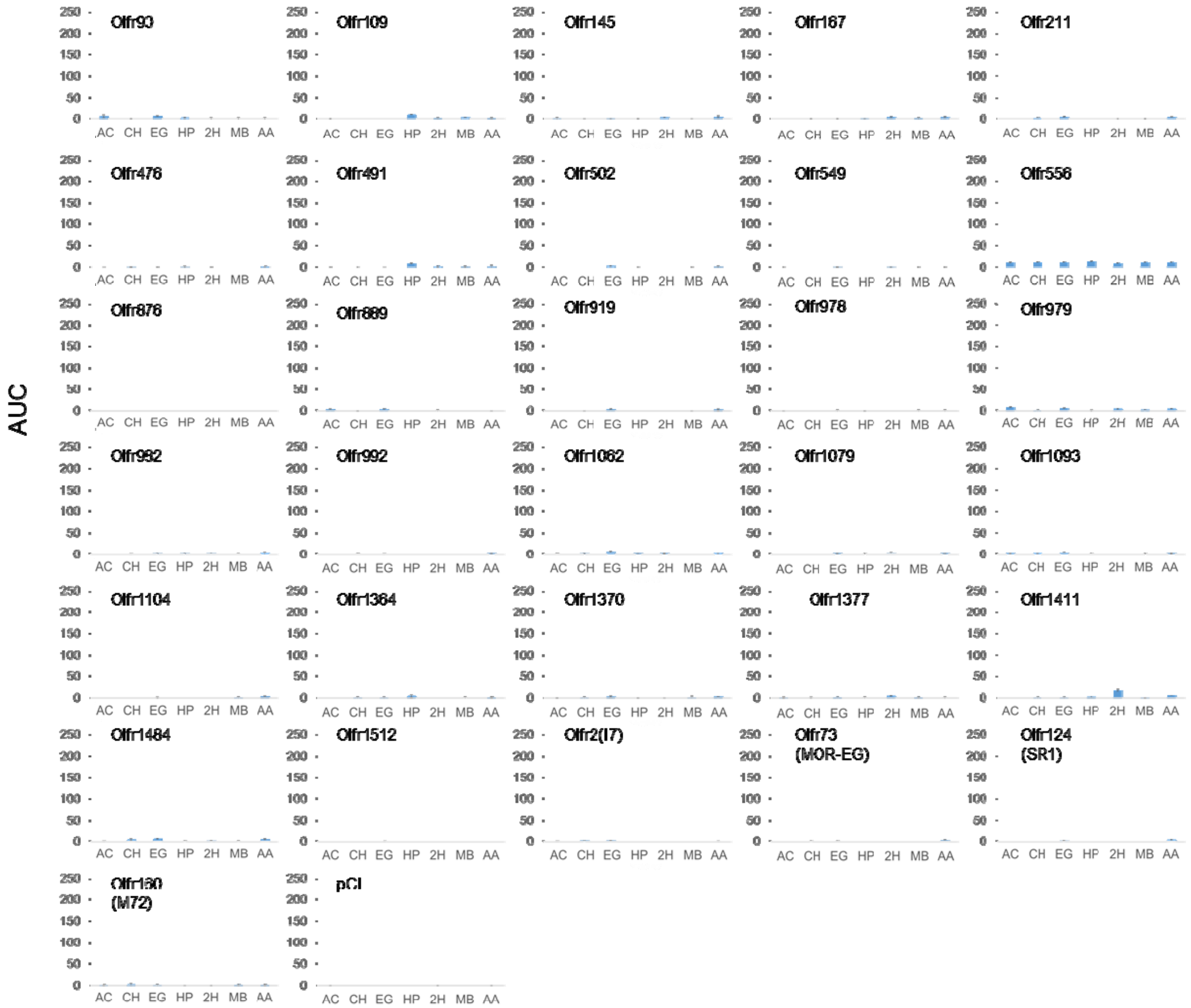

Supplementary Fig. 10
AUC of each OR with stimulated with an odorant at 10^{-8} dilution.
Error bars show s.e.m.(n=3).

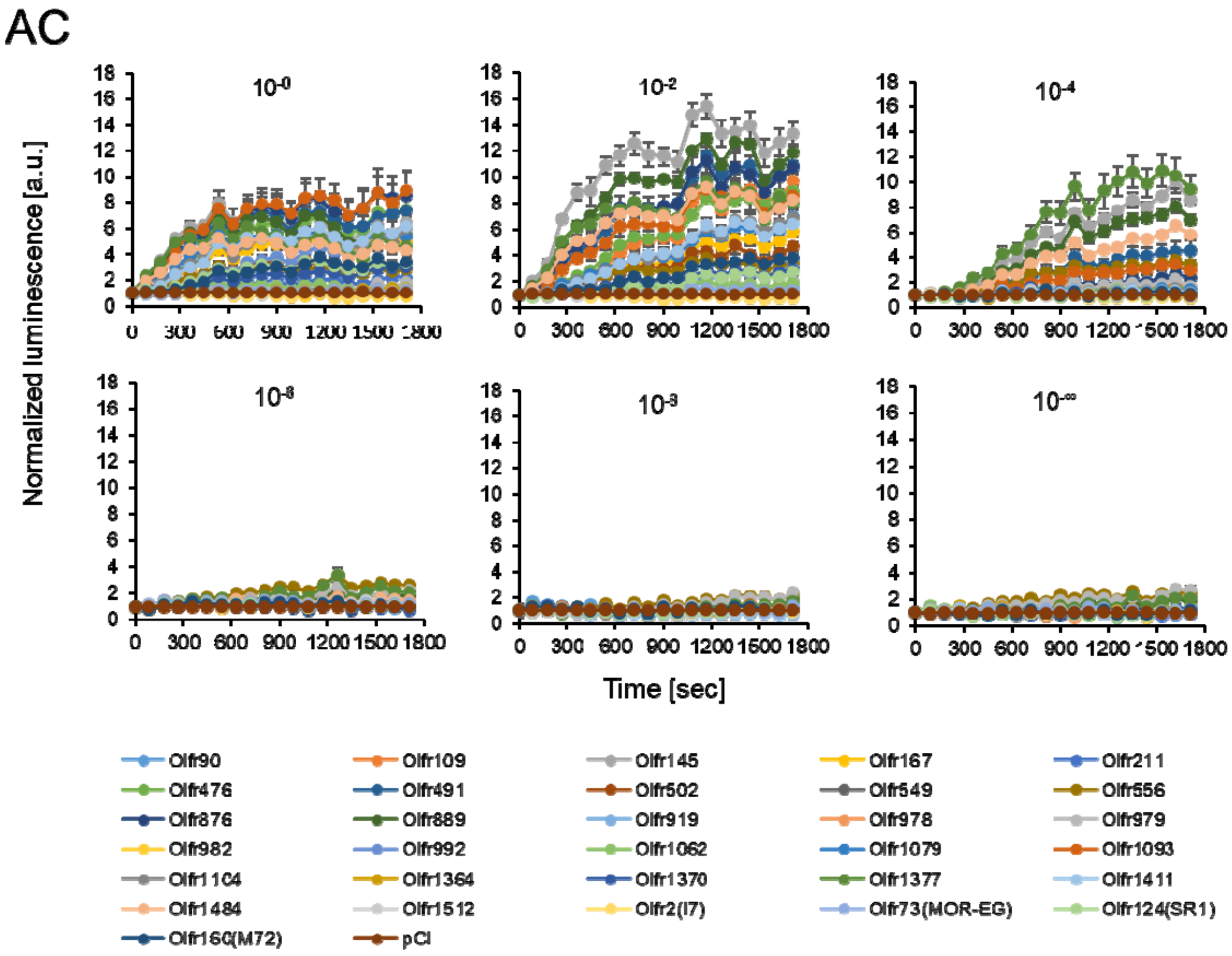

Supplementary Fig. 11
Real time measurement of each OR activation with acetophenone (AC) by Glosensor assay. The luminescence in each well was measured once every 90 seconds, for 20 cycles. The luminescence was normalized such that the initial value of each receptor was defined as 1. Error bars indicate s.e.m (n=3).

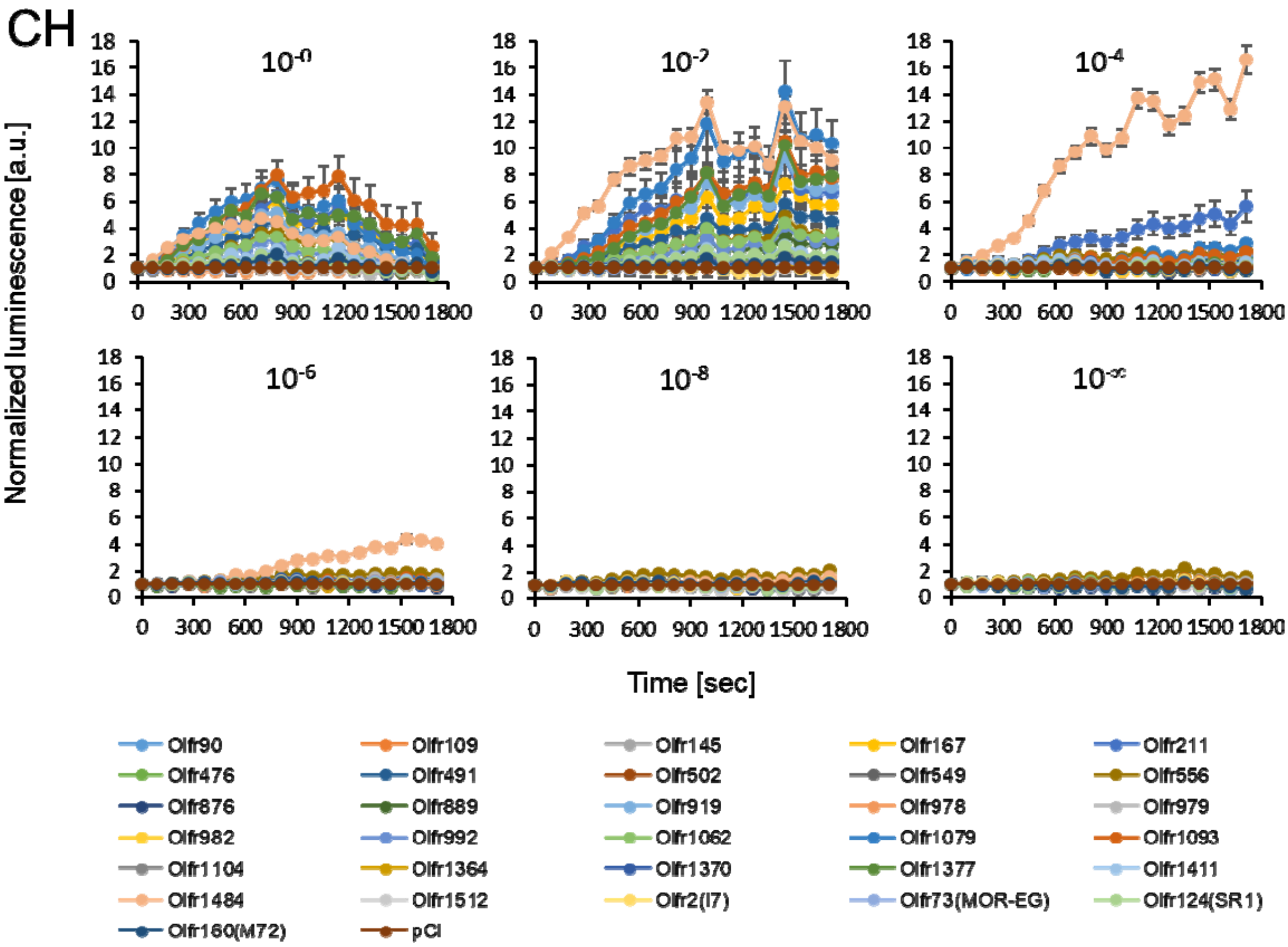

Supplementary Fig. 12
Real time measurement of each OR activation with cyclohexanone (CH) by Glosensor assay. The luminescence in each well was measured once every 90 seconds, for 20 cycles. The luminescence was normalized such that the initial value of each receptor was defined as 1. Error bars indicate s.e.m (n=3).

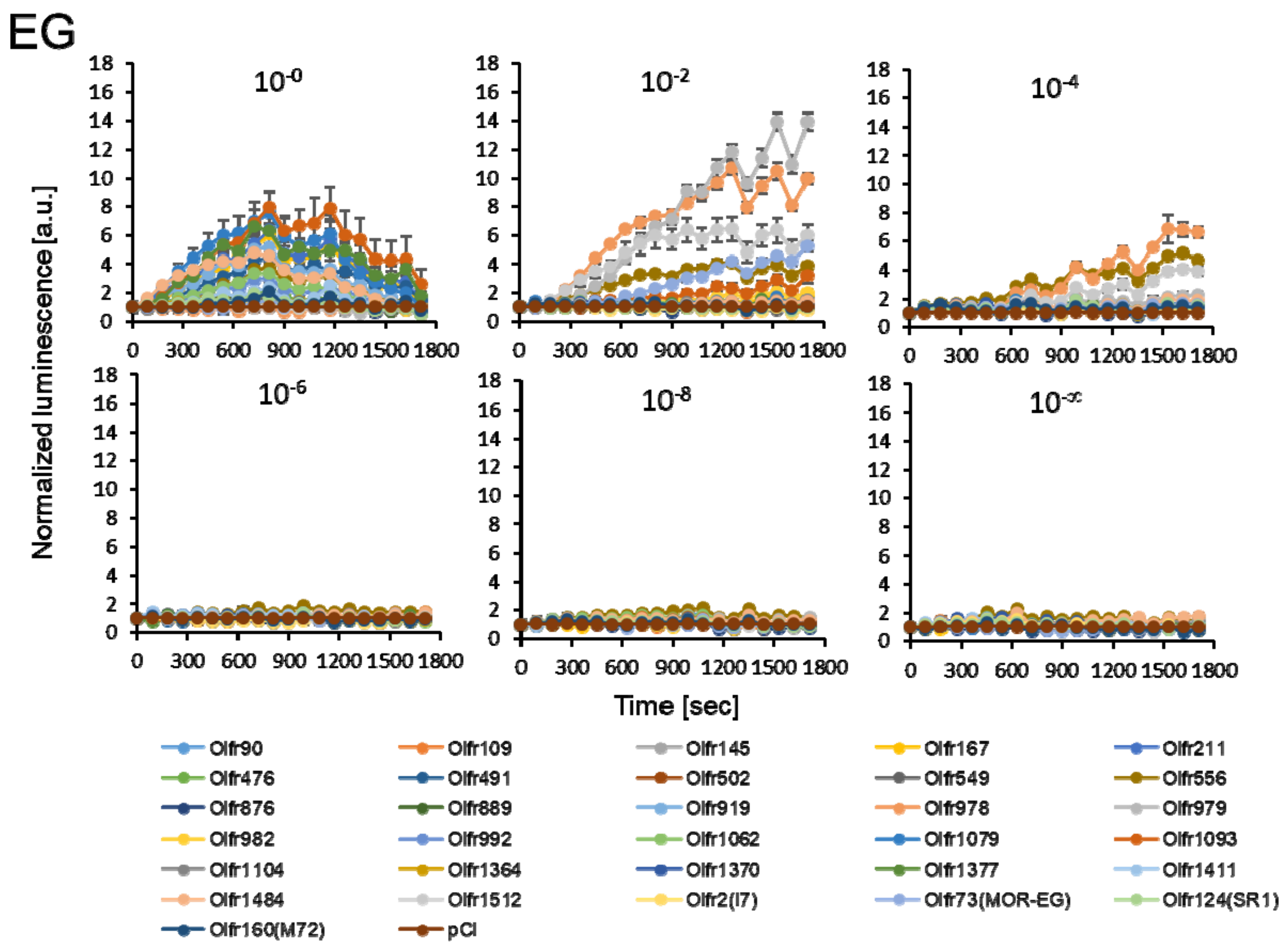

Supplementary Fig. 13

Real time measurement of each OR activation with eugenol (EG) by Glosensor assay. The luminescence in each well was measured once every 90 seconds, for 20 cycles. The luminescence was normalized such that the initial value of each receptor was defined as 1. Error bars indicate s.e.m (n=3).

HP

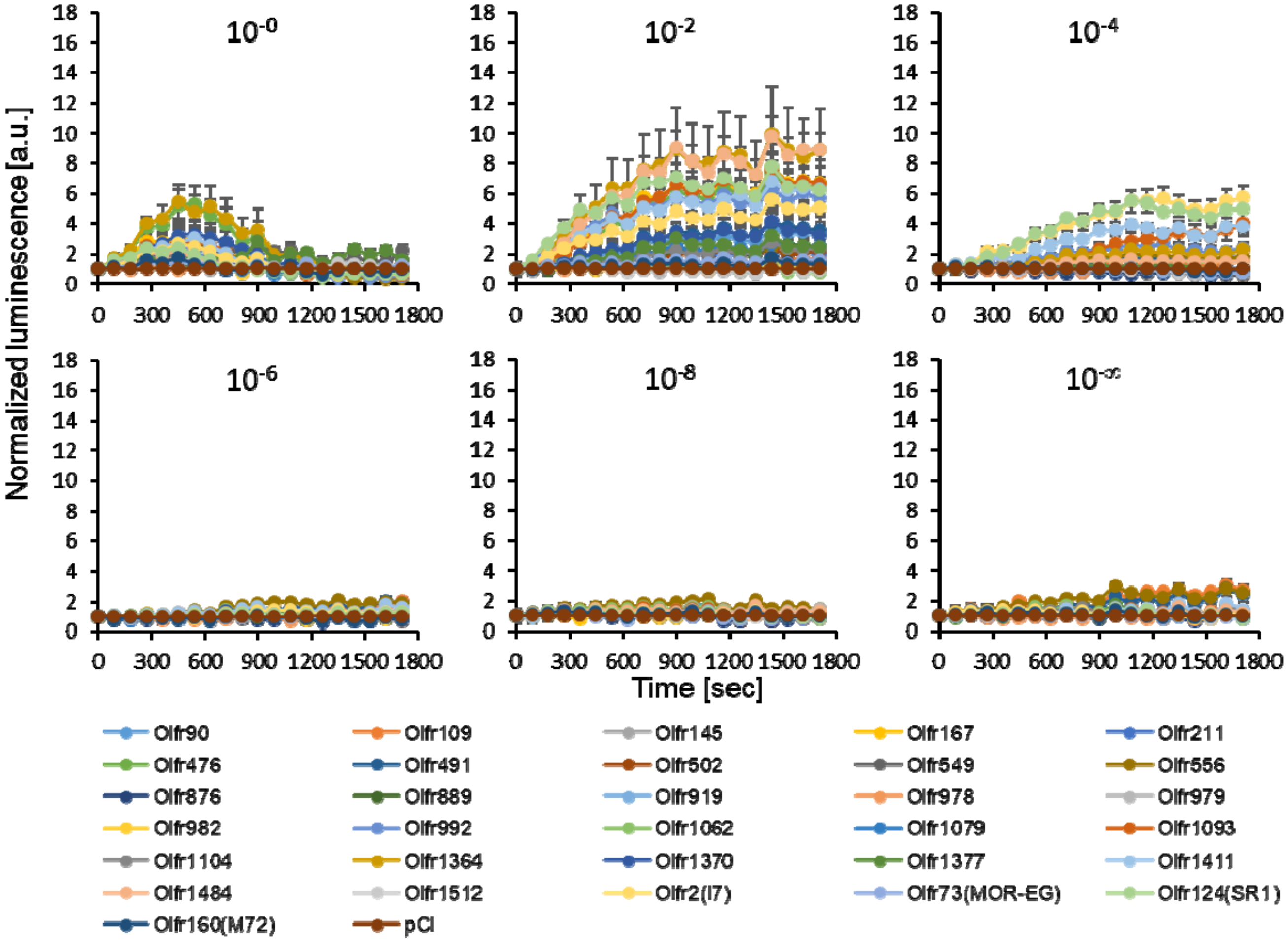

Supplementary Fig. 14
Real time measurement of each OR activation with heptanal (HP) by Glosensor assay. The luminescence in each well was measured once every 90 seconds, for 20 cycles. The luminescence was normalized such that the initial value of each receptor was defined as 1. Error bars indicate s.e.m (n=3).

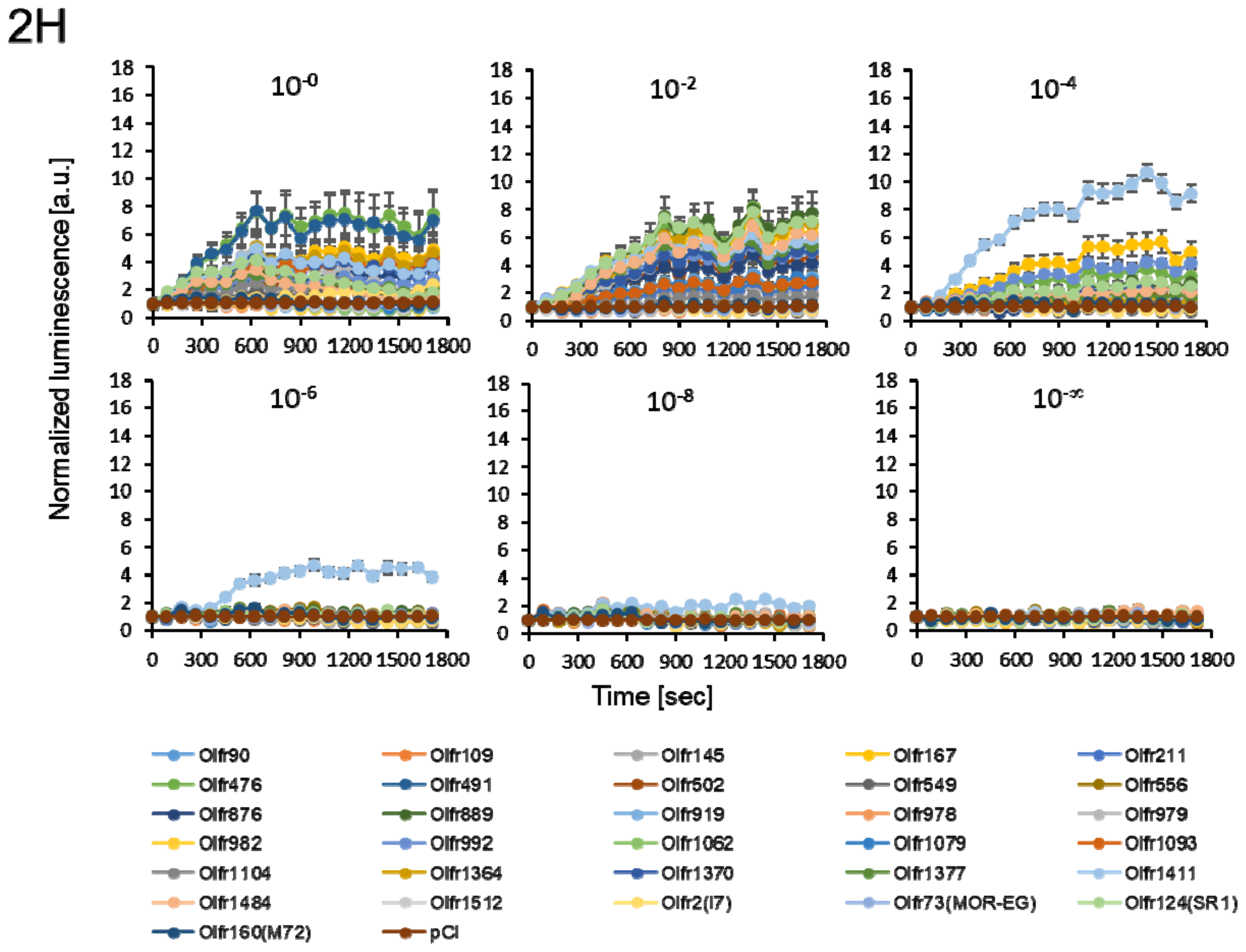

Supplementary Fig. 15

Real time measurement of each OR activation with 2-heptanone (2H) by Glosensor assay. The luminescence in each well was measured once every 90 seconds, for 20 cycles. The luminescence was normalized such that the initial value of each receptor was defined as 1. Error bars indicate s.e.m (n=3).

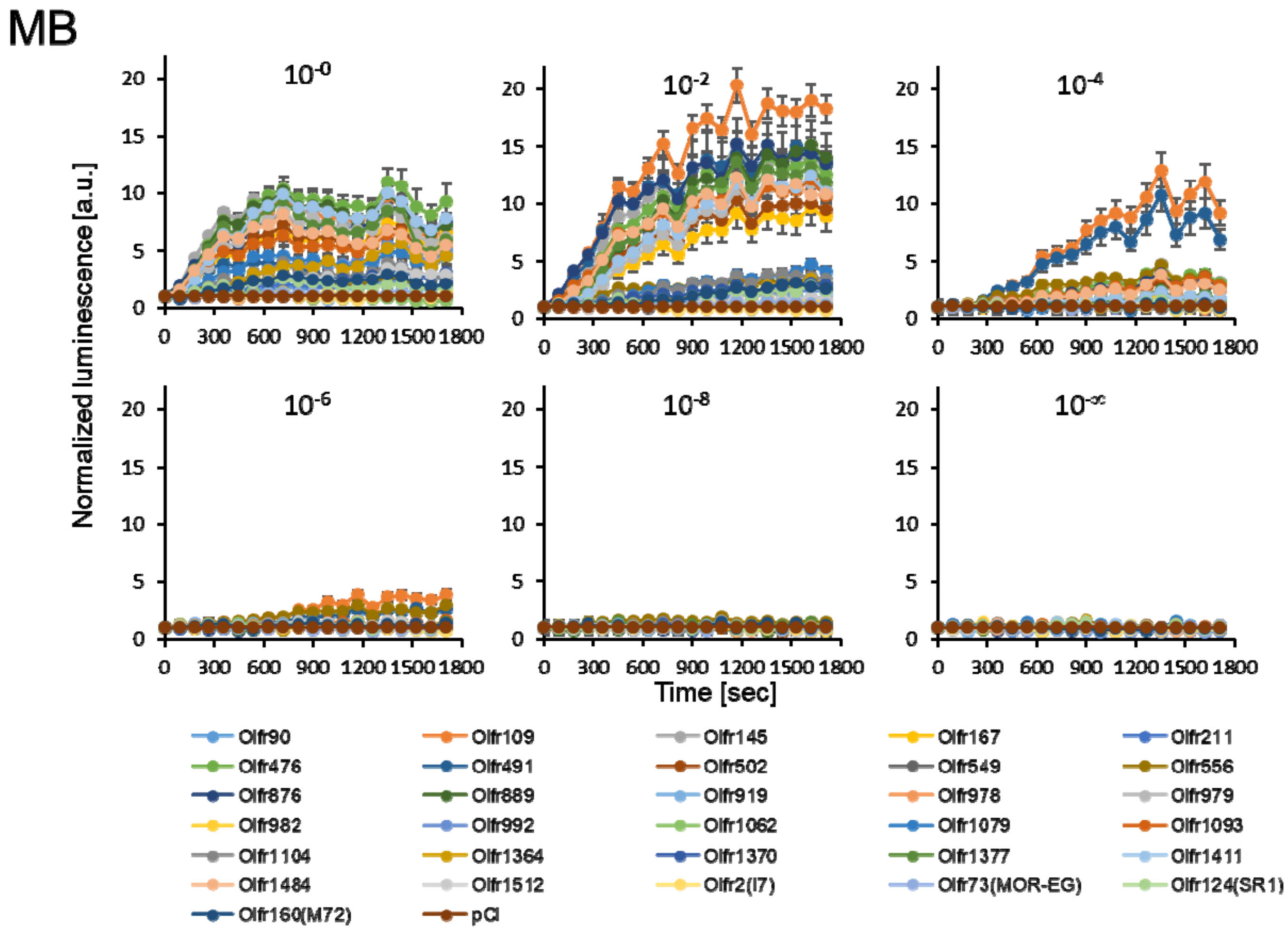

Supplementary Fig. 16
Real time measurement of each OR activation with methyl benzoate (MB) by Glosensor assay. The luminescence in each well was measured once every 90 seconds, for 20 cycles. The luminescence was normalized such that the initial value of each receptor was defined as 1. Error bars indicate s.e.m (n=3).

AA

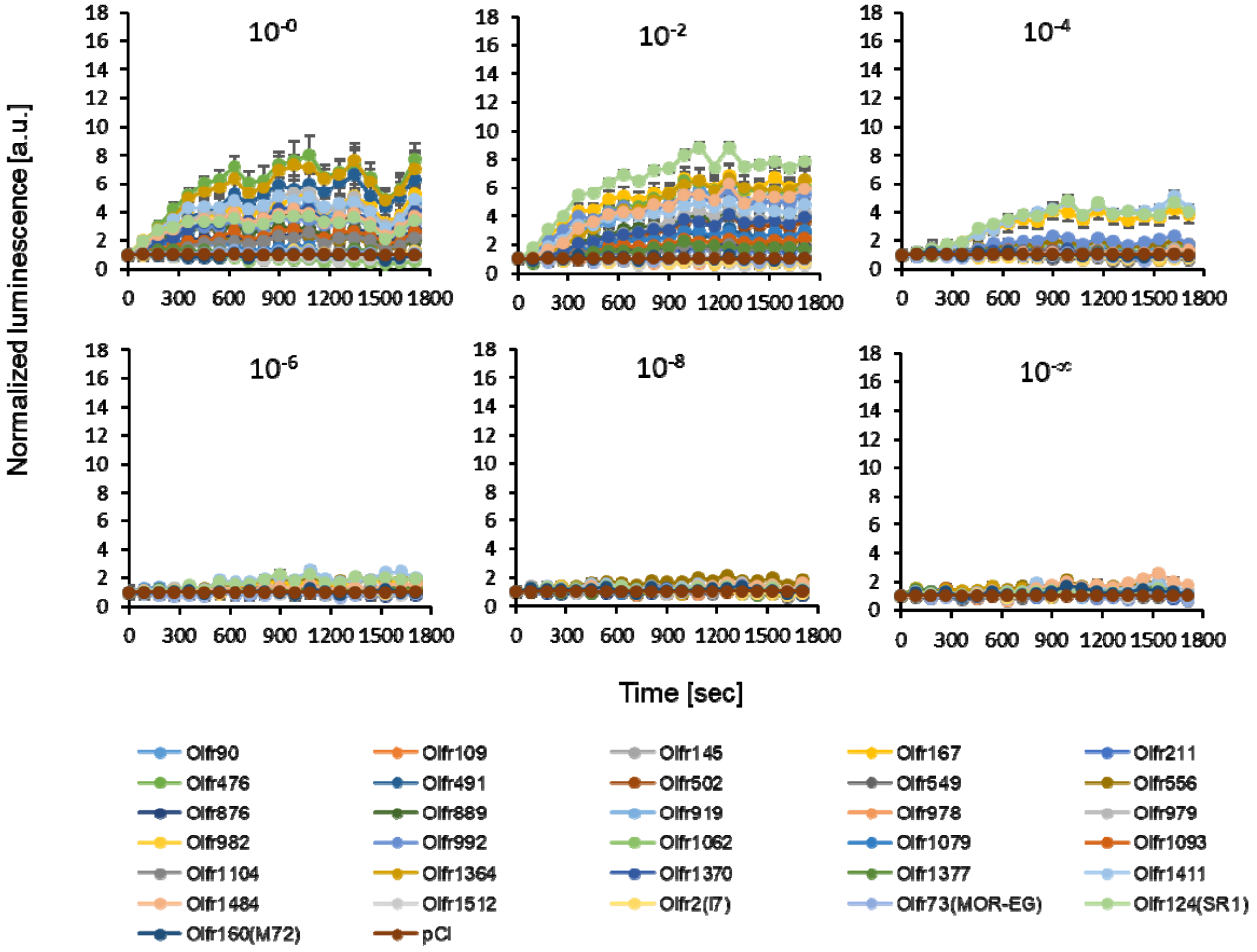

Supplementary Fig. 17
Real time measurement of each OR activation with N-amyl acetate (AA) by Glosensor assay. The luminescence in each well was measured once every 90 seconds, for 20 cycles. The luminescence was normalized such that the initial value of each receptor was defined as 1. Error bars indicate s.e.m (n=3).

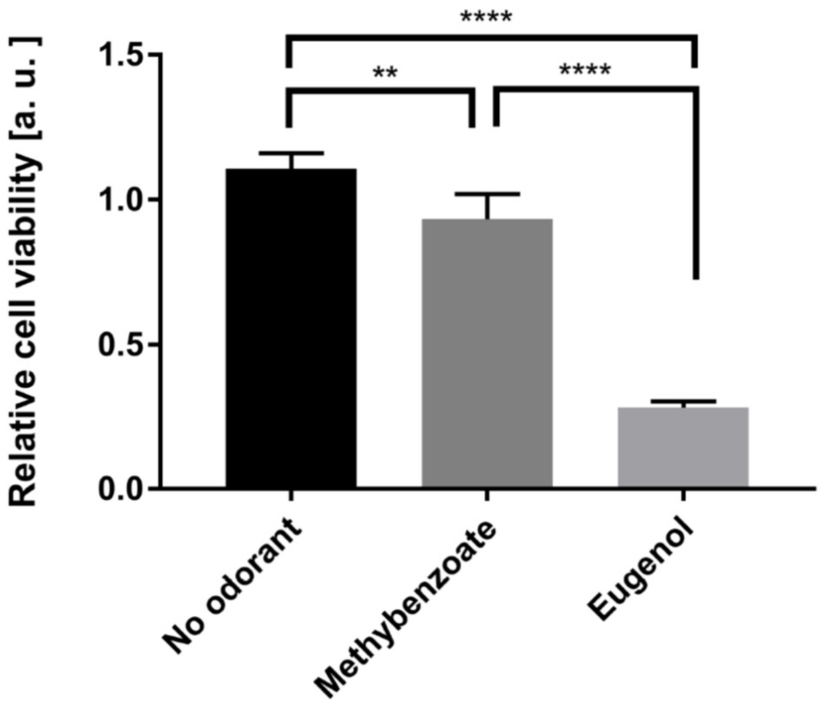

Supplementary Fig. 18
Cell toxicity of odorants exposed to the cells using CellTiter-Glo® Luminescent Cell Viability Assay (Promega). The luminescence in each well was measured at 120 min after stimulation by vapor phase odorant. The values of luminescence were normalized such that the value of each well on before stimulation was defined 1. Error bar indicates s.e.m. (n=4). The p-value was calculated with one-way ANOVA followed by Turkey multiple comparison test (**p<0.01, ****p<0.0001).

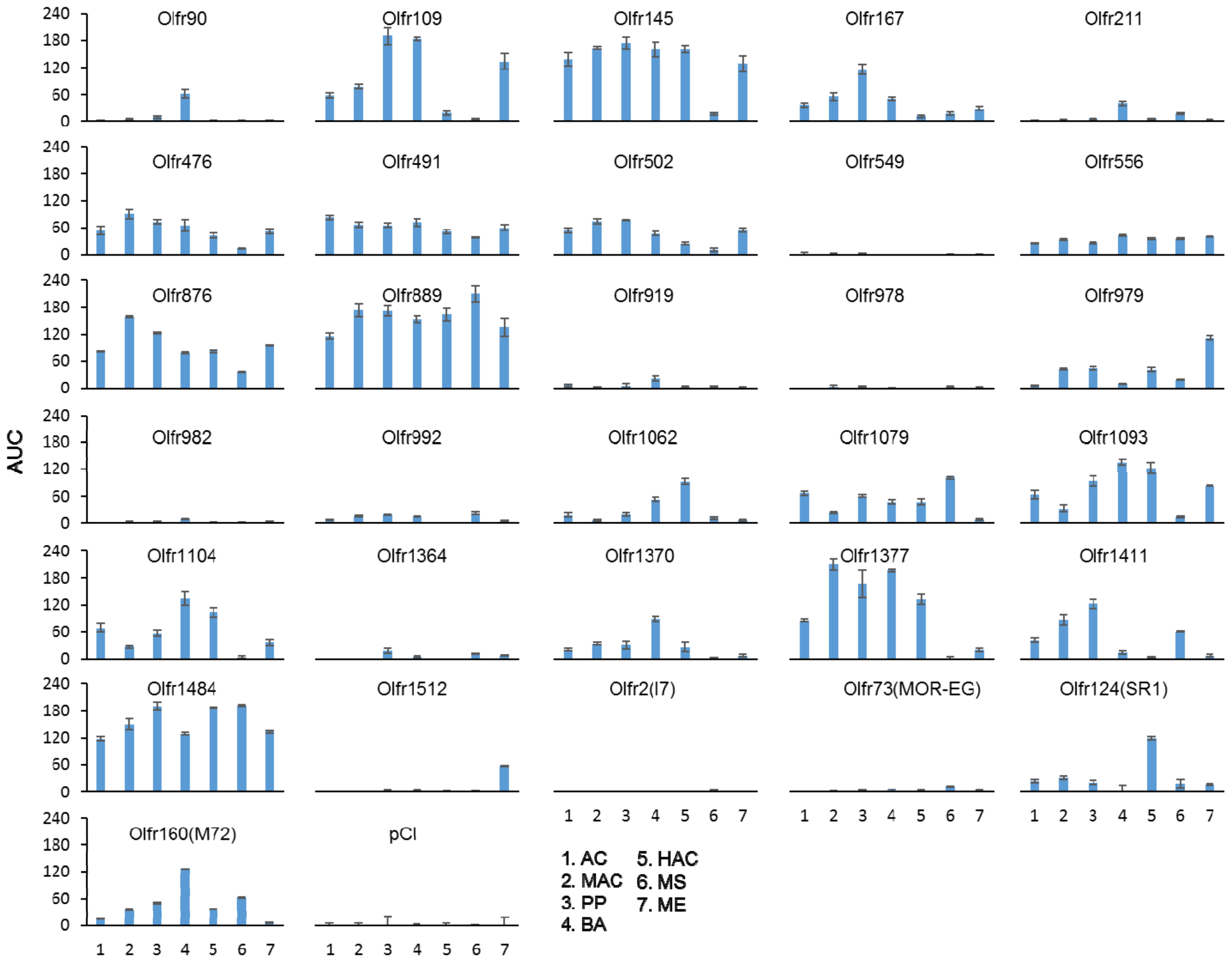

Supplementary Fig. 19

AUC of each OR with stimulated with an odorant at 10^{-2} dilution, which are acetophenone analogs. Error bars show s.e.m.(n=3).

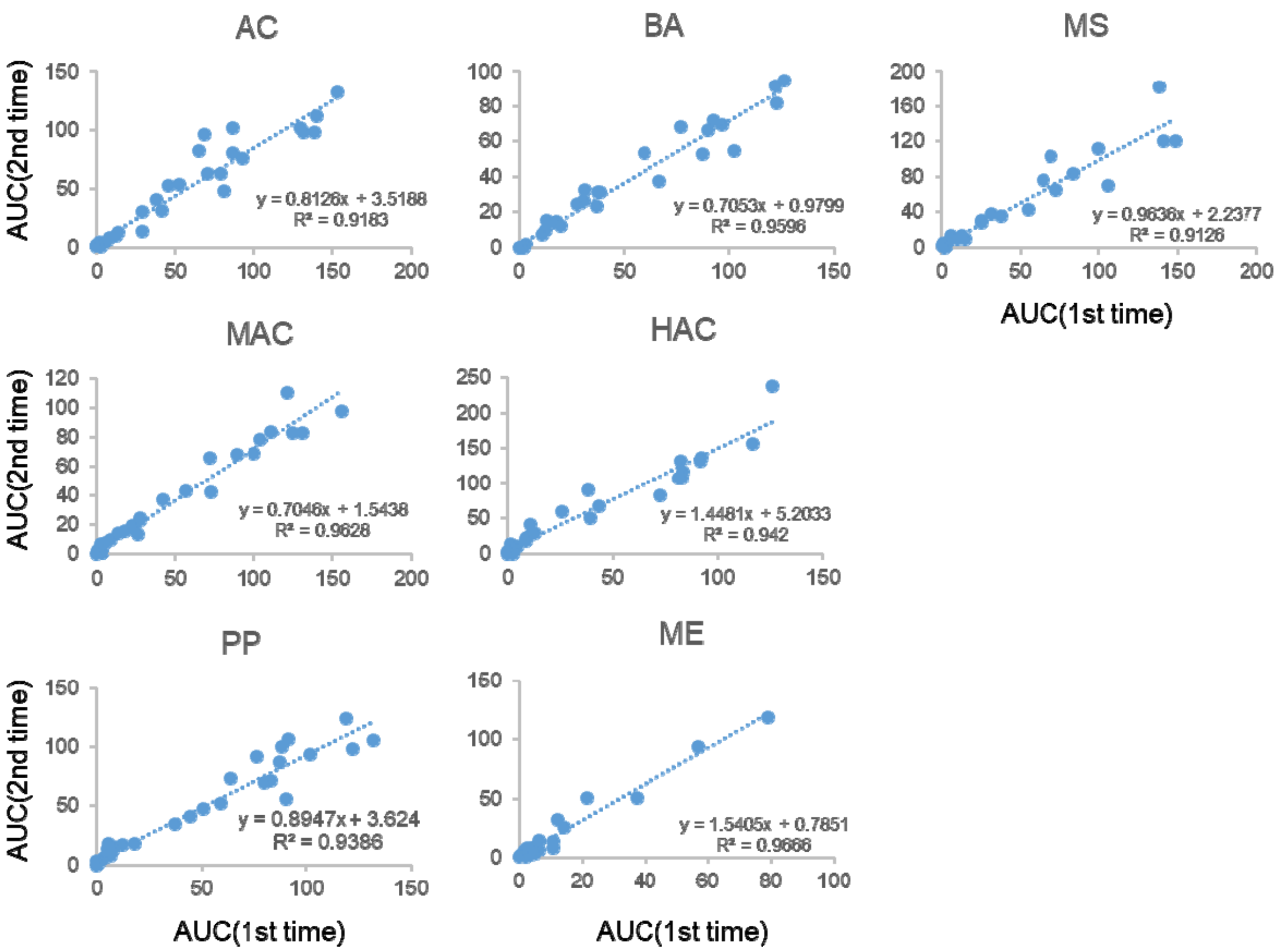

Supplementary Fig. 20
Comparison of separate stimulation by the same odorant using 10^{-2} in acetophenone analogs. R^2 values were calculated by Pearson's correlation coefficient from the linear regression analysis.

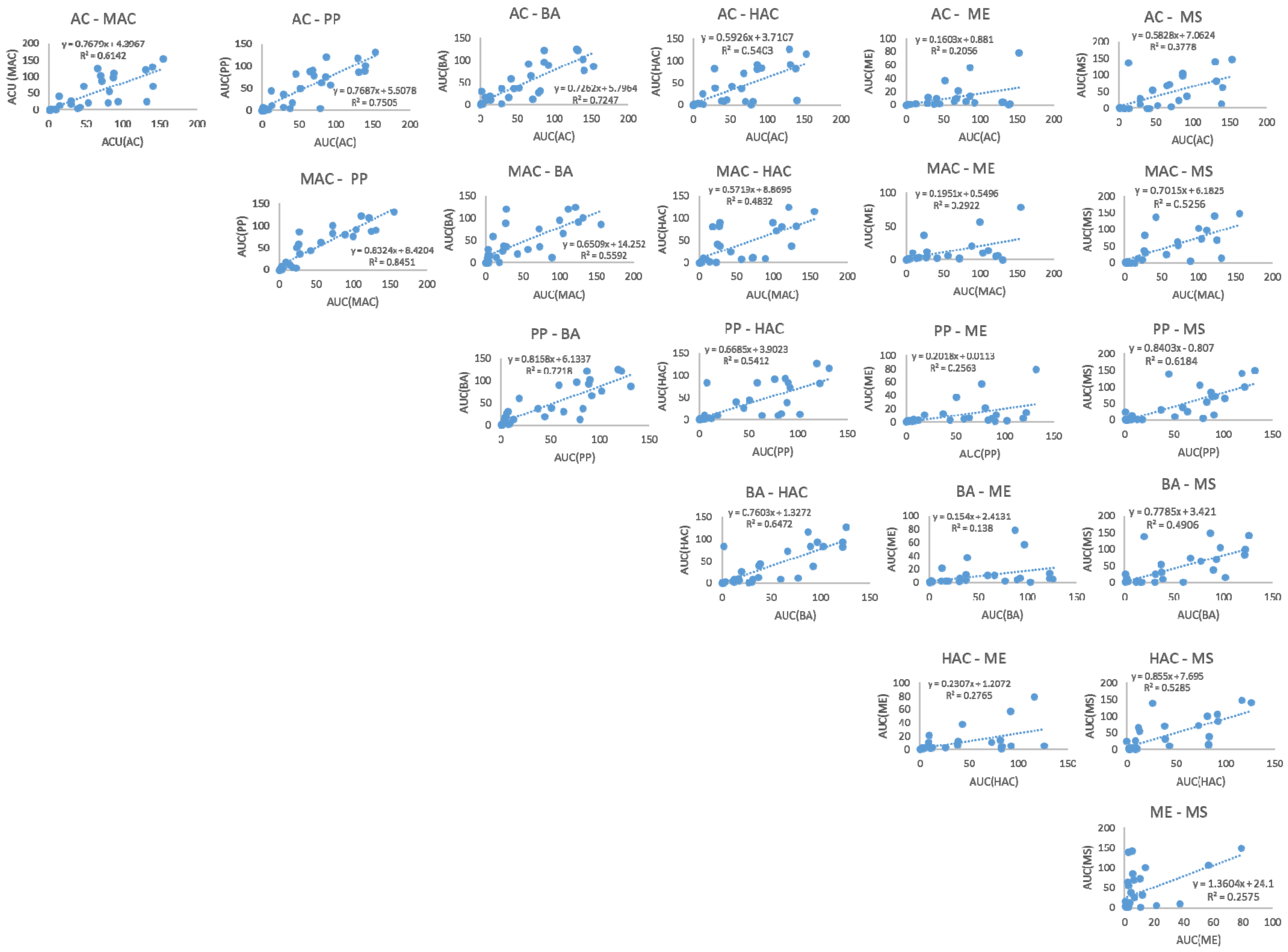

Supplementary Fig. 21
Comparison of separate stimulation by different odorants in Acetophenone analogs. The AUC with vapor was stimulated with an odorant at 10^{-2} dilution. R^2 values were calculated by Pearson's correlation coefficient from the linear regression analysis.

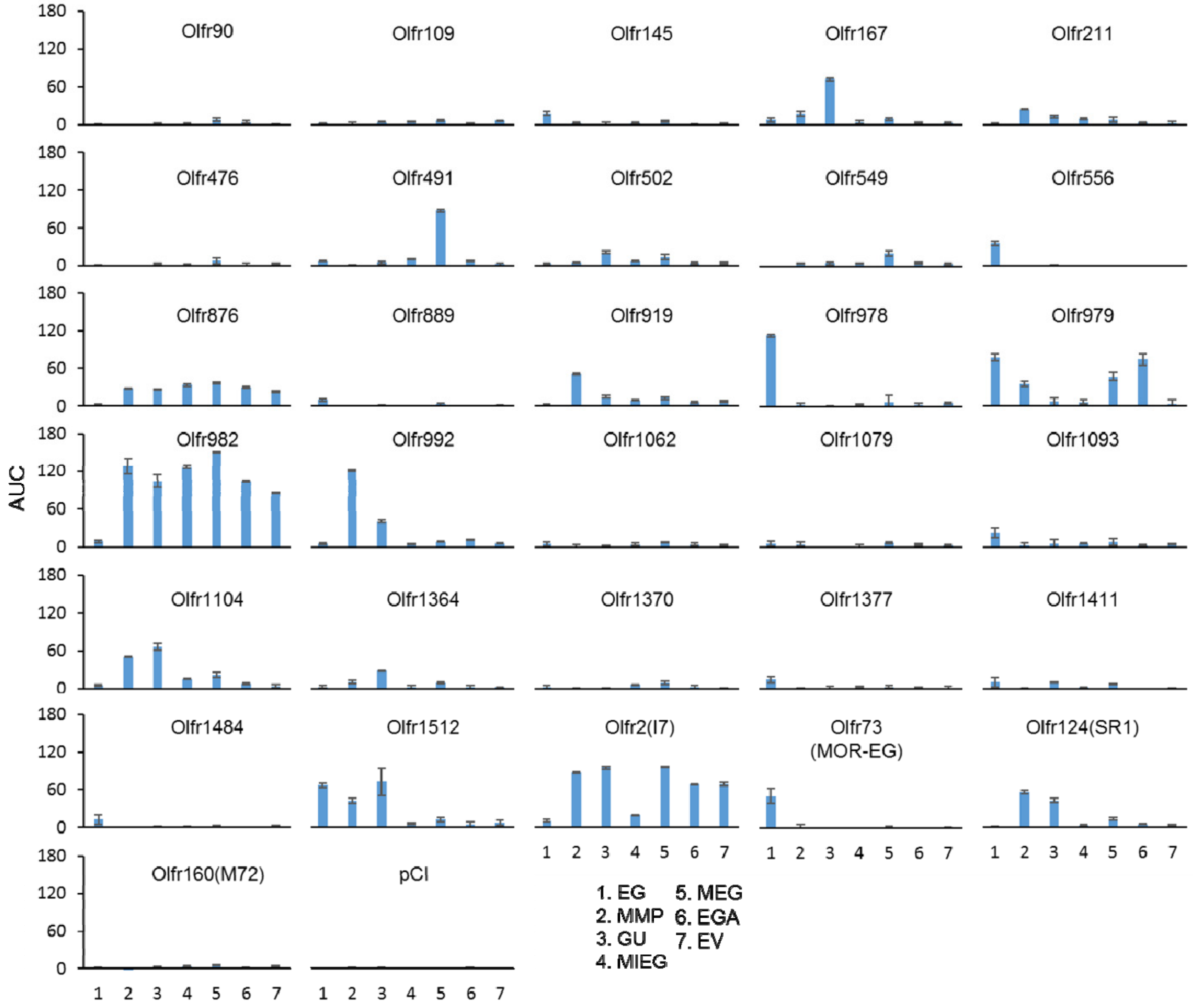

Supplementary Fig. 22
AUC of each OR with stimulated with eugenol analogs at 10^{-2} dilution. Error bars show s.e.m.(n=3).

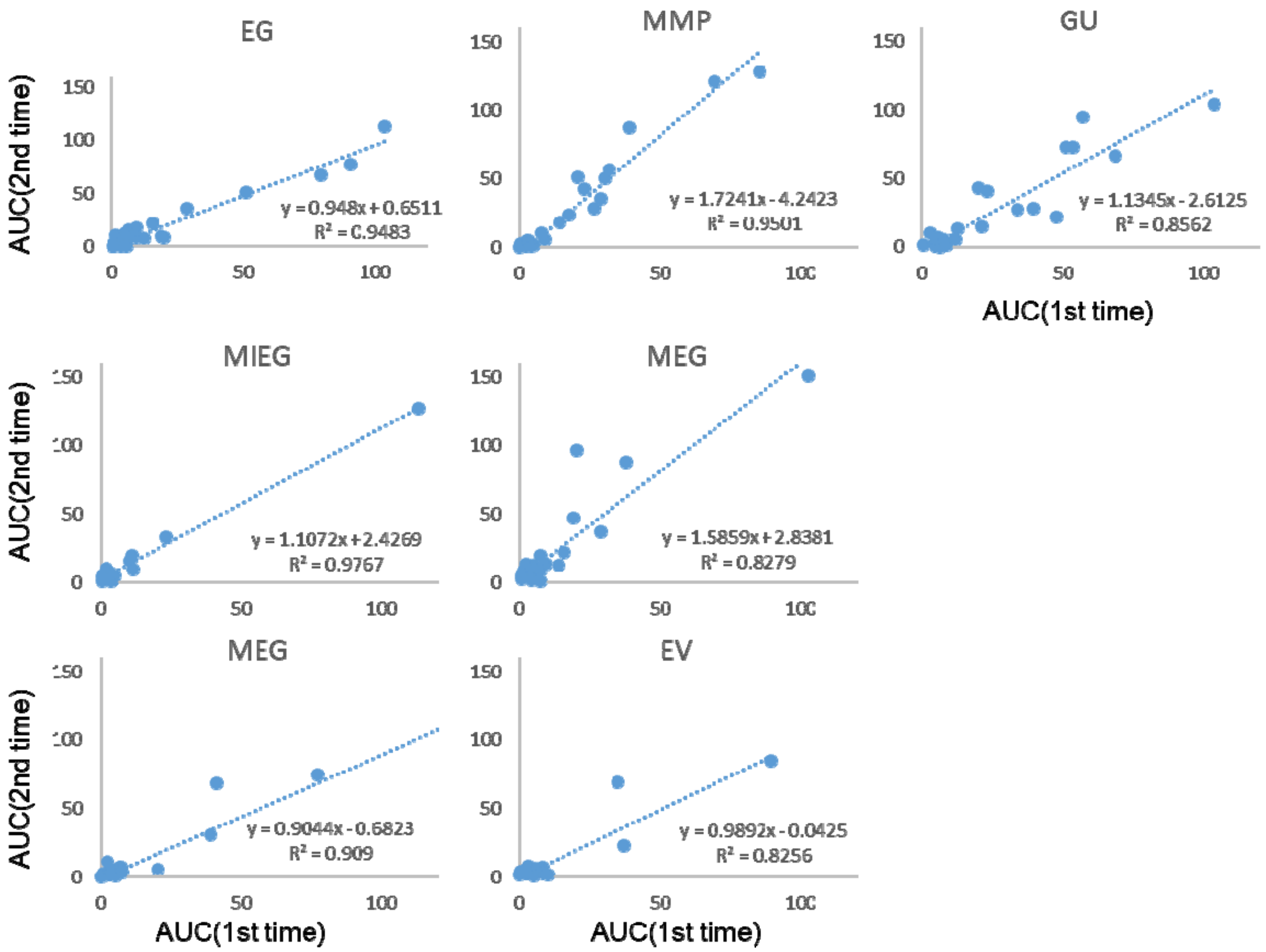

Supplementary Fig. 23
Comparison of separate stimulations by same odorant 10^{-2} eugenol analogs. The AUC with vapor was stimulated with an odorant at 10^{-2} dilution. R^2 values were calculated by Pearson's correlation coefficient from the linear regression analysis.

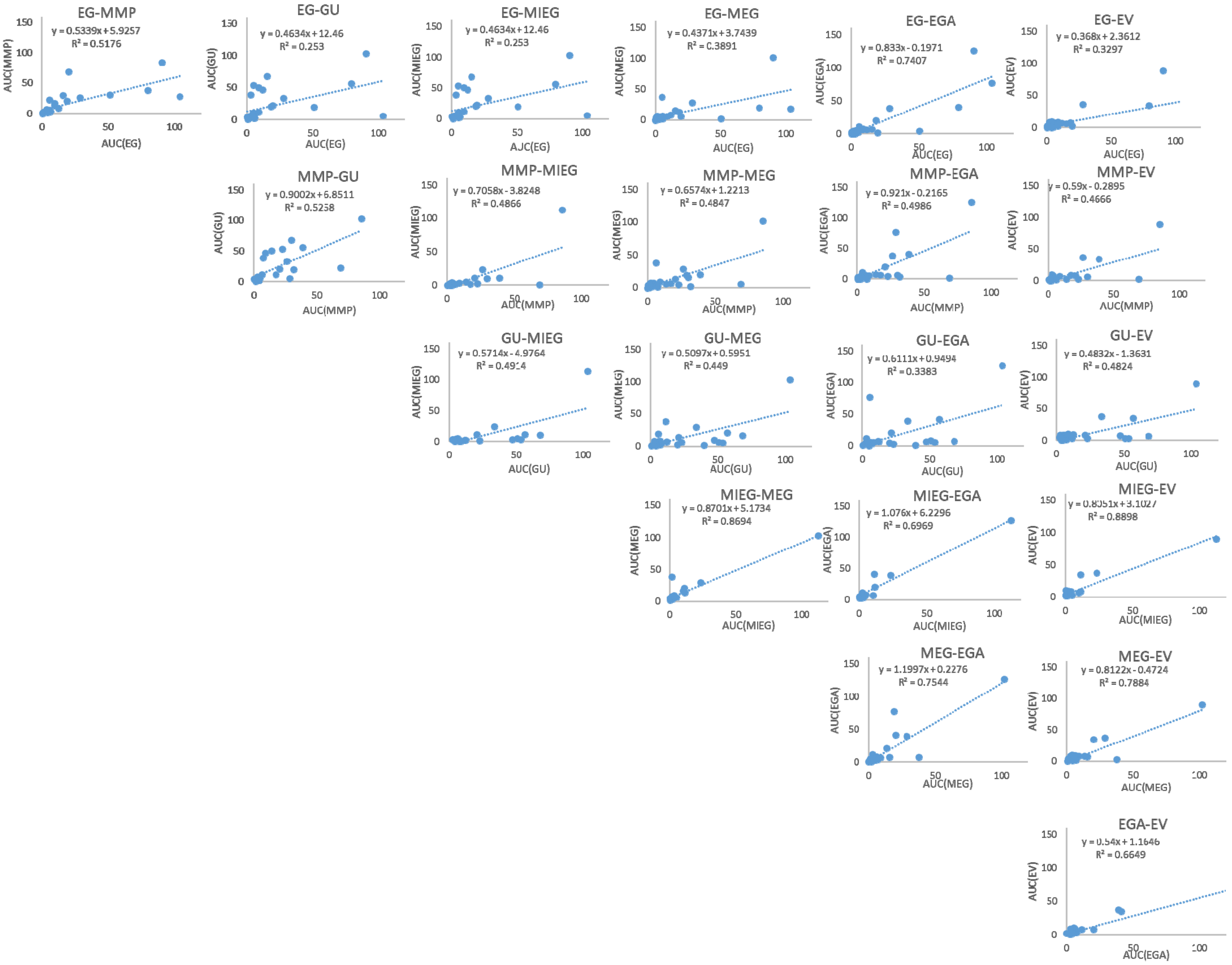

Supplementary Fig. 24

Comparison of separate stimulation by different odorants in eugenol analogues. The AUC with vapor was stimulated with an odorant at 10^{-2} dilution. R^2 values were calculated by Pearson's correlation coefficient from the linear regression analysis.

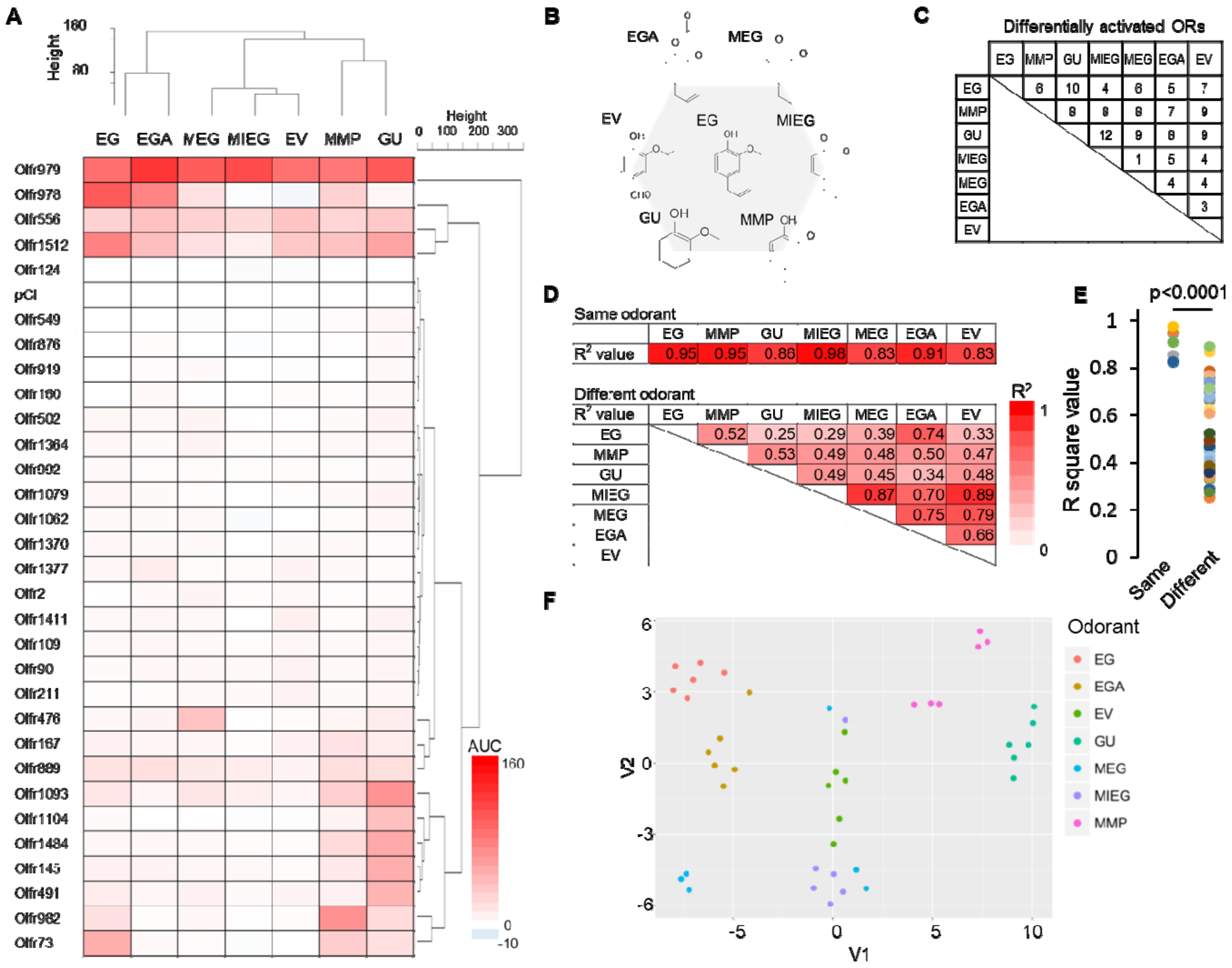

Supplementary Fig. 25

Differential activation of ORs for eugenol analogs. (A) A heat map showing the AUC of each OR stimulated with 10^{-2} odorant. The number on the left side indicates each OR. (B) Chemical structures of each analog used in this assay. Abbreviations for the odorants are as follows: EG; eugenol, MMP; 2-methoxy-4-methyl phenol, GU; guaiacol, MIEG; methyl isoeugenol, MEG; methyl eugenol, EGA; eugenol acetate, EV; ethyl vanillin. (C) The number of ORs that are differentially activated by a tested odorant (Tukey's post hoc analysis, $p<0.05$). (D) Summary of the discrimination analysis using the same and different odorants. Each of R^2 values is shown in Supplementary Figures S23 and S24 (E) Analysis of significant difference between same and different odorants. Student's t-test was performed with R^2 values in Supplementary Fig. 24D. (F) the 32-dimensional space (31 ORs and a vector control) visualized using t-SNE. Each point is an odor with color representing the odor presented and saturation representing whether the point was used to train the model or was part of the test set.

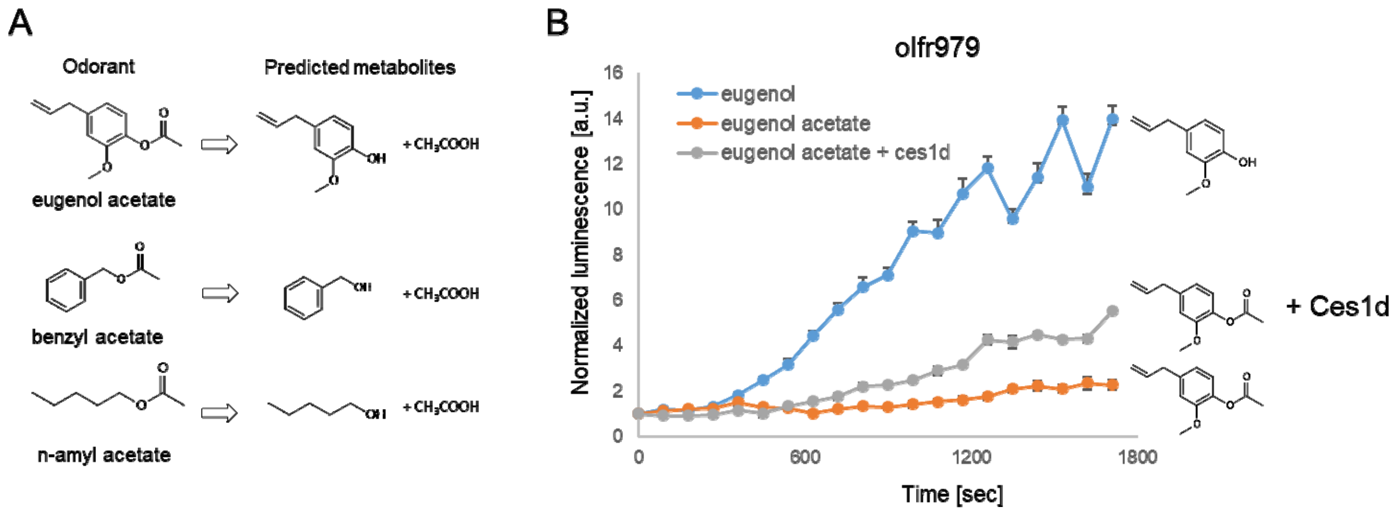

Supplementary Fig. 26
(A) Chemical structures predicted from the enzymatic activity of Ces1d. (B) Olfr979 responds to eugenol (blue), but not to eugenol acetate (Red). Olfr979 co-expressed with Ces1d showed response to eugenol acetate (Gray). Error bar indicates s.e.m. (n=3)

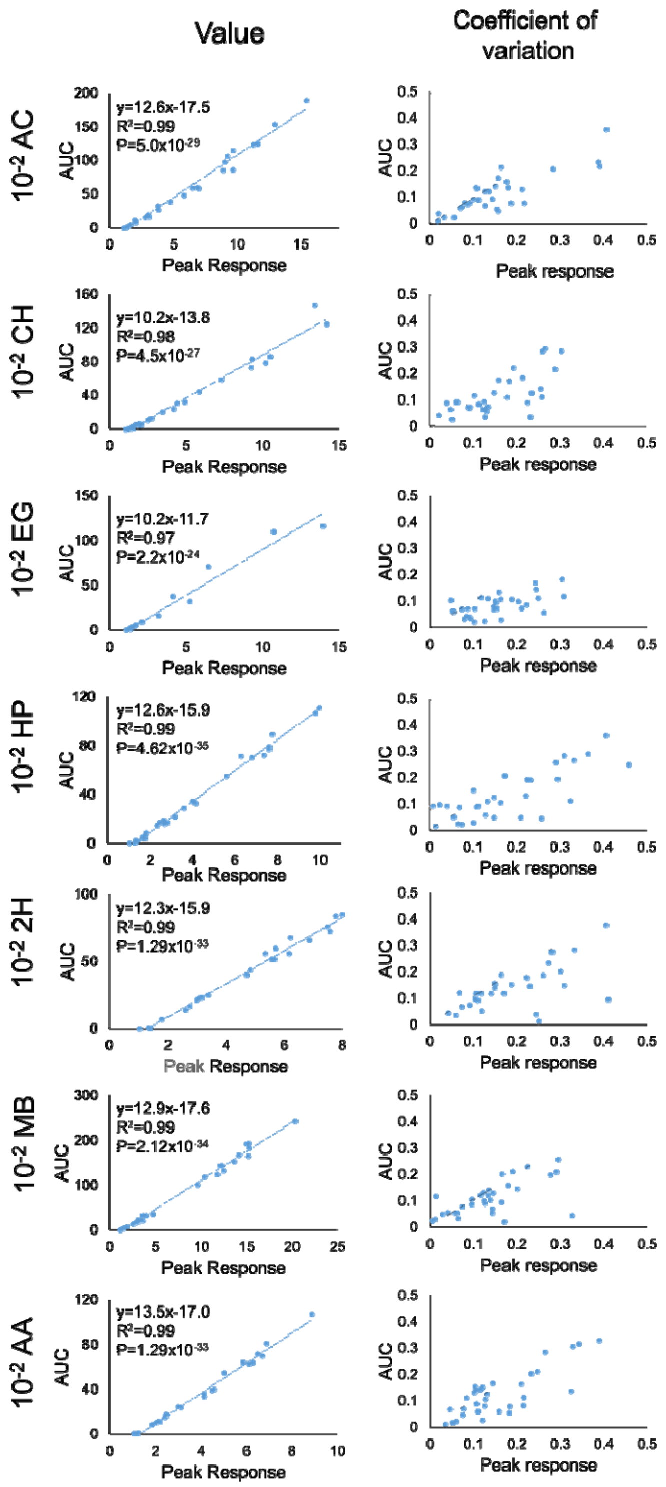

Supplementary Fig. 27

(Left) Comparison of the peak respons and AUC values in the analysis of the receptor response against 10^{-2} of each odorant. The straight line (blue dot) is regression line and R^2 indicates Pearson's correlation coefficient. The p-value was calculated by t-test. (Right) Comparison of the coefficient of variation between peak response and AUC value on 10^{-2} odor stimulation. The black straight line indicates x = y. Coefficient of variation of AUC tend to be lower than that of the peak value.

Supplementary Table 1
Estimated saturated vapor concentration

Estimated saturated vapor concentration from undiluted odorant [ppm]

acetophenone	522
cyclohexanone	6579
eugenol	29.1
heptanal	4631
2-heptanone	5066
methyl benzoate	500
N-amyl acetate	4605

Supplementary Table 2
ANOVA-Dunnett for more frequent odor measurement

ANOVA-Dunnett for more frequent odor mesurment

		olfr145
ANOVA	F value	22.9
	F boundary value	2.95785
	P value	0.00000000008
Dunnett's post hoc	15 sec	0.80712
	30 sec	0.86792
	45 sec	0.14941
	60 sec	0.01661
	75 sec	0.00820
	90 sec	0.00060
	105 sec	0.00106
	120 sec	0.00060
	135 sec	0.00086
	150 sec	0.00015
	165 sec	0.00060
	180 sec	0.00004
