# Supplementary material for: Vapor detection and discrimination with a panel of odorant receptors
Source: Nat Commun. 2018 Nov 1;9:4556. doi: 10.1038/s41467-018-06806-w (PMC6212438; doi:10.1038/s41467-018-06806-w)
Supplement: Supplementary file 2 — Description of Additional Supplementary Files [file 41467_2018_6806_MOESM2_ESM.docx]

**Description of Additional Supplementary Files**

File Name: Supplementary Data 1

Description: Large scale screening of mouse ORs

File Name: Supplementary Data 2

Description: Raw traces (liquid stimulation) with 7 odorants

File Name: Supplementary Data 3

Description: Raw traces (vapor stimulation) with 7 odorants

File Name: Supplementary Data 4

Description: ANOVA and Tukey post hoc tests at 10-2 dilution

File Name: Supplementary Data 5

Description: Raw traces of AC and EG analogous

File Name: Supplementary Data 6

Description: ANOVA and Tukey tests with AC analogous

File Name: Supplementary Data 7

Description: Classifier command lines

File Name: Supplementary Data 8

Description: ANOVA and Tukey tests with EG analogous

File Name: Supplementary Data 9

Description: Raw traces with Ces1d co-expression

File Name: Supplementary Data 10

Description: ANOVA and FDR values with Ces1d co-expression

File Name: Supplementary Data 10

Description: Comparisons between AUC values and Peak responses
